# Reassessing the two-stage theory of social learning development: a discussion

**DOI:** 10.3389/fpsyg.2025.1576714

**Published:** 2025-07-15

**Authors:** Yaming Shang, Yaoru Xia, Yating Yin, Da Dong

**Affiliations:** ^1^Department of Mathematics, Shaoxing University, Shaoxing, China; ^2^Department of Psychology, Shaoxing University, Shaoxing, China; ^3^Center for Brain, Mind and Education, Shaoxing University, Shaoxing, China

**Keywords:** social learning, two-stage development theory, pedagogical clues, selective learning, educational practice, pluralist approach

## Abstract

This article reevaluates the two-stage development theory of social learning by examining the distinct roles of pedagogical and selective learning. Pedagogical learning involves infants’ sensitivity to adults’ communicative intentions, while selective learning highlights how infants prioritize information from reliable sources. Over time, the influence of pedagogical learning diminishes, whereas selective learning continues to play a central role as children grow. We argue for the distinctiveness and plurality of human social learning and emphasize the foundational role of multiple social interactions in cognitive development. Special attention is given to the transition from reliance on pedagogical cues to selective learning, underscoring the critical role of educators in facilitating this shift. We conclude by highlighting the increasing sophistication of cognitive and social selection processes in observation, imitation, and learning, alongside the implications for educational practices. Future research should investigate differences between social and other forms of learning, the mechanisms underlying the diminishing effects of pedagogical cues with age, the cultural variability in social learning trajectories, and the impact of educational technology on the development of social learning.

## 1 Introduction

Social learning, the process of acquiring knowledge through interaction with social entities (e.g., persons), constitutes a central mode of learning that emerges in early childhood (Leadbeater, 2015). In educational psychology, social learning theory explicates how individuals develop skills and understanding by observing and imitating others (Bandura, 1962). This process is marked by the learner’s active engagement, emphasizing their participatory role in acquiring new competencies. A key development in the study of social learning theory has been a shift from a primary focus on pedagogical learning to a more refined exploration of selective learning. Pedagogical learning involves the use of communicative signals, such as gestures or behaviors, that explicitly convey a teaching intent (Ishikawa and Itakura, 2024). Traditionally central to structured educational contexts, pedagogical learning relies on deliberate instruction where learners are guided through systematically designed curricula. This approach has historically provided a foundational framework for organized knowledge transmission in educational settings.

This field has undergone a significant conceptual shift, focusing increasingly on selective learning as a form of autonomous, self-directed engagement, as emphasized by Ishikawa and Itakura (2024). Selective learning conceives individuals as active agents who determine what to learn based on personal interests, goals, and the perceived relevance of information to their development. This perspective moves beyond the passive view of learners as mere recipients of knowledge, reframing them instead as active constructors of understanding within social interactions. The transition from pedagogical learning to selective learning thus marks a reevaluation of the learner’s role, challenging traditional models in which agency rested solely with the teacher or educator. Selective learning highlights how individuals decide which behaviors to imitate and which social signals to internalize, guided by cognitive and emotional appraisal. Research indicates that infants aged 12–15 months selectively imitate actions based on perceived causal necessity. For example, when observing an adult press a light switch with their head, infants typically use their hand instead—demonstrating a cognitive assessment that head-use is nonessential. However, by 15 months, infants begin to exhibit overimitation (Hilbrink et al., 2013). Notably, this form of agency manifests early in infancy—a period foundational to social learning. Even as infants explore the world through observation and imitation, they exhibit striking selectivity, critically discerning which sources of information merit attention. In complex learning environments, infants actively filter and prioritize information, underscoring the pivotal role of social learning in cognitive development. This capacity for discernment equips individuals to identify salient information, fostering adaptability and preparing them for future learning endeavors.

As children develop, their social learning strategies shift from dependence on pedagogical learning to increasingly sophisticated forms of selective learning. Preschool-aged children exhibit the capacity to evaluate the reliability and expertise of others in deciding whom to learn from (Koenig and Sabbagh, 2013). For example, in a classroom setting, a four-year-old might observe Teacher A consistently providing accurate animal facts while Teacher B occasionally offers incorrect information. The child will subsequently prioritize seeking animal-related information from the accurate informant. They also show a deliberate tendency to imitate behaviors consistent with social norms (Harris and Corriveau, 2011), demonstrating advanced selectivity in their learning processes. This capacity is further evidenced by their preference for learning from adults who display knowledge and confidence, their prioritization of accurate information sources, and their inclination to engage in peer learning in contexts where peers may possess greater familiarity (Miosga et al., 2020). These patterns suggest that children adapt their learning strategies contextually, minimizing the risk of misinformation by aligning informational preferences to situational demands.

While social learning theory has illuminated the selective nature of children’s learning, key philosophical questions remain unaddressed. For instance, how do cultural contexts and developmental stages shape the emergence and refinement of selectivity in learning? In what ways does the social environment actively influence these processes? Furthermore, how might educators design pedagogical practices that integrate principles of selective learning to foster children’s epistemic agency and critical engagement? Tackling these questions is crucial for advancing our conceptual understanding of social learning and exploring its practical implications within educational settings.

## 2 Two-stage theory of social learning development

Pedagogical cues, as defined by Csibra and Gergely (2009), refer to an infant’s sensitivity to an adult’s communicative intentions, which can be exemplified by mechanisms such as direct eye contact and the use of infant-directed speech. Selective learning, on the other hand, highlights the ability of infants to acquire knowledge from reliable sources of information (Koenig and Sabbagh, 2013), a skill that is especially crucial in uncertain environments. Research has demonstrated that infants selectively engage in social learning situations, which aids in the processing of information (Baldwin and Moses, 1996). As the child grows, the influence of pedagogical cues tends to wane, while the impact of selective learning remains prominent in older children (Matheson et al., 2013). These developmental differences are believed to arise from the varying utilities of learning strategies in uncertain environments (Legare and Nielsen, 2015). Carpenter et al. (1998), in a longitudinal study of 24 infants aged 9–15 months, documented that social-cognitive skills develop sequentially through sharing, joint attention following, and attention-directing behaviors. This developmental trajectory diverged significantly from non-social skill acquisition, highlighting the foundational role of social interaction in cultural learning. The findings of this research underscore the uniqueness of human social learning and the pivotal role that social interaction plays in cognitive development (Ishikawa and Itakura, 2024; Tomasello and Carpenter, 2007).

We will tackle the development of social learning through the lens of developmental science, highlighting the significance of understanding this process in comprehending how infants learn and adapt within intricate social environments (Baldwin and Moses, 1996; Gottlieb et al., 2013). It posits that from the neonatal stage, infants exhibit a high degree of sensitivity to social stimuli, such as the direct gaze of others. Research indicates that infants younger than 18 months are more inclined to engage in social learning within both instructional and selective learning contexts; however, these facilitating effects may wane or vanish as they develop. For instance, a 20-month-old infant is equally likely to follow the gaze of others, regardless of whether eye contact is present, suggesting a reduction in the influence of apparent signals at this age. This developmental shift demonstrates that children increasingly select information sources based on reliability rather than pedagogical cues as their cognitive abilities mature (Baldwin and Moses, 1996). Additionally, 18-month-old infants demonstrate more imitative behaviors in interactive scenarios compared to 24-month-old infants, while both age groups exhibit similar levels of imitation in both interactive and non-interactive situations. These findings imply that the enhancing effect of pedagogical cues may diminish with maturation.

In contrast, selective learning remains advantageous for older children. Empirical evidence indicates that children aged 4–7 are more disposed to imitate individuals who exhibit greater expertise, suggesting a tendency to learn from those perceived as experts (e.g., Koenig and Sabbagh, 2013). This selective imitation emerges from children’s prioritization of the dominant learning factor—a cognitive focus that shifts from pedagogical cues toward source expertise and reliability. Furthermore, a comprehensive meta-analysis of 63 studies involving 6,525 participants revealed that selective vocabulary learning is observable in children ranging from 2 to 5 years of age. These findings collectively demonstrate that as children mature, they continue to engage in selective learning, a strategy that is underpinned by the knowledge or experience of others, contributes to more efficient learning, and is regulated through top-down cognitive processes.

While social interaction is intrinsically rewarding during development, learning from unreliable or out-group information providers can present significant risks, including exposure to deception or threats. It has been argued that selective learning evolved as a mechanism to mitigate such risks by prioritizing reliable sources of information (Legare and Nielsen, 2015). Empirical studies also suggest that adult gaze-following behaviors are subject to modulation by top-down social factors, such as group membership (Ciardo et al., 2014) and social status (Dalmaso et al., 2012). These findings indicate that socially and epistemically relevant cues shape the dynamics of attention and interaction (Dong and Chen, 2025). Consequently, as development progresses, knowledge-based top-down moderation may enhance the effectiveness of social engagement and learning by enabling individuals to navigate complex social environments more adaptively.

The principles of the two-stage theory of social learning development offer valuable insights for designing more effective learning strategies in educational contexts. By understanding how children transition from reliance on pedagogical cues to selective learning, educators can tailor their teaching methods accordingly. In the early stages of education, when children are particularly sensitive to pedagogical cues, teachers should utilize direct instruction and demonstrations to guide learning effectively. As children mature and their capacity for selective learning develops, educators should shift towards providing diverse learning resources and fostering independent exploration.

Specific strategies educators can implement include incorporating interactive sessions and group discussions to promote active learning and critical thinking. Creating opportunities for students to engage with varied perspectives and critically evaluate information supports the development of their selective learning skills. Furthermore, educators can leverage technology to deliver personalized learning experiences that accommodate individual interests and learning paces. This approach not only enhances learning efficiency but also equips students to navigate the complex and dynamic learning landscapes of the future.

## 3 The primacy of pedagogical clues

The interactive patterns of the caregiver-infant dyad have a profound impact on the infant’s brain development and learning capabilities (Chen et al., 2024; Ilyka et al., 2021). During the early childhood social learning phase, pedagogical cues are central to guiding individual learning behaviors (Csibra and Gergely, 2009). These cues, which can manifest as direct instructions, demonstrations, or signals conveyed through speech, body language, and facial expressions, assist children in comprehending when, where, and how to learn (Gergely and Csibra, 2006). In their formative years, children’s educational experiences are significantly influenced by the guidance they receive from adults, which aids them in navigating their environment and acquiring new knowledge. For instance, explicit instructions from parents and teachers, such as “look at me, do as I do,” serve as pedagogical cues that prompt children to imitate and learn. This clear direction helps children establish foundational behavioral patterns and cognitive frameworks (Csibra and Gergely, 2009), setting the stage for their subsequent independent learning.

However, as children’s cognitive abilities and environmental adaptation enhance, the role of pedagogical cues gradually diminishes, giving way to sensitivity to more complex cues (Koenig and Sabbagh, 2013). Stahl and Feigenson (2015) demonstrated that 11-month-old infants treat violations of their expectations as unique learning opportunities. When observing events that contradict their knowledge, these infants exhibit enhanced learning, deeper exploration, and specific hypothesis-testing behaviors. This transition signifies a shift from pedagogical learning to selective learning, where children begin to determine whether to imitate or learn based on various factors, including the reliability of the information source, the skills of the demonstrator, and their own needs. This shift reflects children’s burgeoning initiative and discernment in social interactions; they are no longer passive imitators but active agents capable of dynamically adapting their learning strategies to their own needs and environment. However, the pedagogical clues did not disappear in the process, but instead changed their role, moving from direct instructions to supportive signals. They continue to guide children to understand learning contexts, but more often by providing structures and frameworks to help children make selective learning decisions from a wider range of resources. The role of the educator also shifts at this stage, from being a direct guide to a facilitator, encouraging children to think independently and explore independently, thereby promoting the development of their selective learning.

From an educational practice standpoint, this transition holds significant implications. As children mature, their reliance on directive instruction decreases, while their need for autonomy in shaping their learning preferences and methods increases. This developmental shift stems from children’s growing capacity to prioritize the most critical learning factor: information source credibility and relevance. Consequently, the traditional one-size-fits-all approach to education is increasingly obsolete, educators must shift their approach from merely imparting knowledge to facilitating the discovery of knowledge, the evaluation of information, and the exercise of choice. For instance, in the classroom, teachers can incorporate more interactive sessions and group discussions, providing children with opportunities to articulate their own, assess the views of others, and make informed learning decisions based on these exchanges. Creating learning spaces that promote autonomy and self-directed growth enables students to develop critical adaptive competencies for a dynamic global landscape.

Moreover, educators can leverage a diverse array of teaching and learning resources, such as multimedia materials, field trips, and project-based learning, to create rich learning environments that stimulate children’s interest and initiative in learning. Simultaneously, this shift underscores the importance of cultivating children’s selective learning skills through the creation of supportive educational environments that encourage exploration, experimentation, and the acceptance of mistakes. For example, parents can foster a culture of inquiry by encouraging their children to pose questions in daily life and guiding them to seek answers independently, rather than providing them directly. Schools can also offer a variety of courses and activities that children can select based on their interests and abilities, thereby nurturing their self-directed learning and decision-making skills. Nonetheless, it must be recognized that disparities in resource accessibility persist among students. Socioeconomic differences exert a substantial effect on the quality of educational opportunities afforded to children. To rectify this inequity, effective collaboration between schools and policymakers is necessary to guarantee that all students, irrespective of background, have equitable access to a broad range of learning resources and developmental opportunities.

## 4 The transition from pedagogical clues to selective learning

The evolution of social learning is a gradual and complex process rather than a linear progression, with the most significant transformation being the shift from reliance on pedagogical cues to the development of selective learning strategies (Koenig and Sabbagh, 2013). This developmental transition typically emerges during infancy, as infants become increasingly attuned to environmental cues and begin to engage in selective imitation and learning behaviors (Baldwin and Moses, 1996). Bandura’s (1977) social learning theory further elucidates this process, positing that individuals cognitively process observed behaviors, evaluate their potential consequences, and subsequently determine whether to replicate them. Importantly, this selective capacity is not innate but rather develops progressively through environmental interactions and social experiences.

During early childhood, pedagogical strategies, particularly those involving demonstrations and guidance from caregivers or educators, play a pivotal role in facilitating cognitive development (Bjorklund et al., 1997). These strategies provide explicit learning models that assist children in comprehending appropriate behaviors and their contextual applications. As children’s cognitive abilities and social experiences expand, their information-processing mechanisms undergo significant transformation. They transition from passive recipients of pedagogical cues to active evaluators and selectors of learning sources. This developmental shift aligns with Piaget’s (1954) conceptualization of “formal operational thinking,” characterized by the emergence of abstract reasoning capabilities and the ability to solve increasingly complex problems. Vygotsky’s (1978) concept of the Zone of Proximal Development (ZPD) further underscores this shift by highlighting the essential role of social interaction and guided learning in cognitive growth. For example, Gehlot (2021) emphasized that the Zone of Proximal Development (ZPD) is dynamical, constantly evolving as learners acquire new knowledge and skills. Through interactions with more knowledgeable others, children internalize new knowledge and skills, gradually progressing from dependence on external guidance to independent learning. Cultivating critical thinking skills early on is therefore more important than ever, particularly during this transition. In an era marked by prevalent misinformation and fake news, the ability to evaluate the credibility and relevance of information is crucial. Consequently, educators should prioritize teaching students how to question, analyze, and synthesize information, moving beyond passive acceptance. This objective can be achieved through structured activities such as debates, research projects, and media literacy education.

For instance, preschool-aged children demonstrate the capacity to differentiate individual competencies in specific tasks through observational learning and selective imitation. They preferentially emulate individuals who exhibit expertise in particular domains rather than indiscriminately imitating all models. This selective learning strategy not only enhances learning efficiency but also mitigates the acquisition of potentially erroneous or suboptimal information, thereby facilitating the advancement of cognitive and social competencies. Poulin-Dubois and Brosseau-Liard (2016) demonstrate that infants show selective imitation based on perceived competence: 14-month-olds preferentially imitate adults who demonstrate object proficiency (e.g., correctly versus incorrectly putting on shoes), while 18-month-olds decline to imitate actions or learn labels from inaccurate informants (e.g., speakers who misname objects).

The transition from reliance on pedagogical cues to selective learning is a multifaceted process influenced by numerous factors. Primarily, cognitive development plays a pivotal role; for example, the maturation of working memory enables children to process increasingly complex information, while enhanced executive function facilitates decision-making and self-regulation (Doebel et al., 2016). Secondly, the social environment, particularly familial and educational contexts, significantly shapes the development of selective learning. Supportive environments that encourage exploration and experimentation are particularly conducive to fostering selective learning capabilities. Research demonstrates that a caregiver’s presence signals safety, reduces children’s fear, and enables exploration of challenging environments. This security allows children to investigate negative cues (e.g., shapes paired with unpleasant sounds), directly facilitating selective learning (Gopnik, 2019). Additionally, cultural context exerts considerable influence, as diverse cultural frameworks present distinct learning paradigms and value systems that inform children’s selection of learning resources (Chen, 2012).

Educators play a crucial role in facilitating this developmental transition (Harris and Corriveau, 2011). They should recognize and accommodate children’s emerging selective learning tendencies by providing diverse and challenging learning materials that stimulate active exploration. Furthermore, educators should cultivate secure learning environments that encourage independent thinking while simultaneously developing children’s critical thinking and self-regulatory capacities. Through such pedagogical approaches, children can not only optimize learning efficiency but also cultivate the sophisticated skills necessary for adapting to future societal requirements. Teachers frequently implement inquiry-based learning techniques to motivate students to formulate their own questions and independently discover answers. This approach fosters the development of selective attention and autonomous learning capabilities (Bell et al., 2005). Moreover, integrating collaborative learning activities helps children build essential social skills while promoting peer knowledge exchange. Such collaboration strengthens students’ ability to critically evaluate and apply knowledge, complementing inquiry-based methods to foster comprehensive development (Johnson and Johnson, 1999).

The ontogeny of social learning represents a developmental progression from dependence on pedagogical cues to the emergence of selective learning strategies, reflecting both cognitive maturation and enhanced social adaptability. Educators and researchers should adapt pedagogical approaches based on this developmental understanding to support holistic child development, thereby establishing a robust foundation for subsequent learning and life experiences. Future research should deepen our understanding of the core processes driving this developmental shift while examining how diverse cultural and educational environments shape selective learning across contexts. Such insights will inform the refinement of educational practices and guide the creation of effective learning strategies for all children globally.

## 5 Concluding remarks: a multifaceted process?

Since Shulman’s foundational work in the 1980s and 1990s, Pedagogical Content Knowledge (PCK) has evolved to incorporate technological dimensions, resulting in the “Technological Pedagogical Content Knowledge” (TPCK/TPACK) framework. This model, which underscores the intricate knowledge structure required for teachers to effectively integrate technology into teaching, has become a cornerstone of educational technology theory (Mishra and Koehler, 2006). The framework has significantly influenced the development of technical competencies in teacher professional development, particularly in the realms of online education and blended learning. Pedagogy, defined as the art and science of teaching, informs teachers’ actions, judgments, and instructional strategies (Loughran, 2013). Loughran further advocated for pedagogy rooted in reflective practice, a perspective that has shaped the “practice-oriented” approach in teacher education. These theoretical foundations have enriched empirical research on teachers’ classroom behaviors. For example, a 2023 study investigated the correlation between teachers’ instructional practices and the reduction of student anxiety, conceptualizing “pedagogical behavior” as the synthesis of teachers’ knowledge, patience, feedback, personal characteristics, and teaching strategies (Asare, 2023). Gökçek and Yılmaz (2019) emphasized the integration of pedagogical skills with both face-to-face and distance teaching strategies, highlighting the importance of aligning technological tools with educational objectives. Similarly, Susanto et al. (2020) defined pedagogical competency as the ability to design collaborative learning experiences and create contextualized teaching environments, thereby fostering social interaction and practical application in teacher education.

In the realm of educational technology, a hybrid strategy combining recommendation algorithms with learning style recognition has been proposed as a core technical framework for personalized learning platforms (Klašnja-Milićević et al., 2011). The widespread citation of this approach underscores its significant impact on areas such as course recommendation and learning path optimization, where it has markedly improved learning efficiency. Hwang et al. (2012) further validated the influence of learning styles on outcomes through the development of adaptive games. Gamified learning strategies, particularly in STEM education, have since gained traction for their ability to enhance student engagement and knowledge retention by dynamically adjusting game difficulty and content. Spector’s (2014) Intelligent Learning Environment (ILE) framework, which integrates adaptive technology, big data analytics, and learning analytics, has provided a theoretical foundation for the design of personalized educational systems. This framework has catalyzed a shift from static learning environments to dynamic, adaptive models. However, research has also revealed that selective categorization in machine learning, such as the rejection of certain predictions, can exacerbate inequalities among different groups. These findings have drawn academic attention to algorithmic bias, prompting the development of fairer models and establishing ethical review as a critical component of algorithm design.

With the proliferation of online education and blended learning models, traditional pedagogical approaches have become increasingly inadequate in addressing the diverse learning needs of children. Selective learning, by contrast, offers a more adaptable framework for these evolving educational paradigms, enabling children to autonomously navigate and select learning pathways from an extensive array of resources. This approach emphasizes children’s agency and initiative, fostering the development of independent thinking and problem-solving skills—competencies that are indispensable for future educational and professional success, as they equip children to make informed decisions in complex and dynamic environments. The TPACK framework, introduced by Mishra and Koehler (2006), highlights the intricate knowledge structure required for teachers to effectively integrate technology into instruction, thereby advancing technical competencies in teacher professional development. As educational technology continues to evolve, children must also learn to selectively utilize technological tools and resources to adapt to varied learning contexts. Spector’s (2014) theoretical framework of intelligent learning environments, which integrates adaptive technology, big data analytics, and learning analytics, provides a robust theoretical foundation for the personalized design of educational systems. Such advanced learning environments facilitate the transition from traditional, one-size-fits-all approaches to targeted learning by delivering tailored content and feedback aligned with each child’s educational progress and performance.

In this article, we highlight a transition from reliance on pedagogical cues in infancy to the refined selection of information sources in childhood. This shift not only reflects the continuity of cognitive development but also underscores the potential influence of educational practices on this process. The critical role of pedagogical cues in early childhood education, coupled with the profound impact of selective learning on children’s long-term development, necessitates that educators and psychologists understand this transition. Educational strategies must be sufficiently flexible to accommodate the evolving learning needs of children. Research on the relationship between domain-general learning abilities and infant selective social learning reveals no significant correlation, suggesting that social learning operates independently of domain-general learning mechanisms (Crivello et al., 2018; Crivello and Poulin-Dubois, 2019; Luchkina et al., 2018). The dynamic nature of social learning highlights the progressive enhancement of individuals’ initiative and decision-making skills within social contexts. Consequently, educators must transition from being mere transmitters of information to assuming roles as guides and facilitators of learning (Daouk et al., 2016). By offering a diverse range of learning resources and fostering a supportive learning environment, educators can effectively nurture children’s selective learning abilities, thereby enhancing learning efficiency, minimizing the impact of irrelevant information, and cultivating critical thinking and self-regulation skills. These competencies lay a solid foundation for lifelong learning and adaptability in an ever-changing world.

Besides, the pivotal role of educators in children’s learning and development highlights the imperative for them to transition from traditional roles as mere knowledge providers to multifaceted roles as guides, facilitators, and architects of the learning environment. The findings accentuate the significant impact of cultural context on learning strategies (Nisbet et al., 2005), suggesting that educational approaches within the globalization framework should emphasize cultural adaptability and diversity. In today’s globalized world, cultural competence transcends mere desirability to become a necessity. As such, students need to develop the skills to navigate diverse cultures and perspectives. This imperative requires educators to integrate multicultural education into curricula, actively exploring the richness of varied cultural traditions. Cultivating a classroom environment that acknowledges and values cultural diversity is equally imperative. This ensures that each student is recognized as worthy of respect, regardless of their background. Concurrently, the rapid advancement of science and technology underscores the integration of educational technology into the learning process as a cornerstone of educational innovation. This integration necessitates a collaborative effort among educators, psychologists, and technology developers to explore the utilization of new technologies in enhancing the learning experience and improving efficiency.

Moreover, more and more findings robustly endorse the student-centered education paradigm, advocating for an educational focus on individual differences and unique needs. This approach entails providing tailored learning pathways and support to foster students’ self-directed learning capabilities, critical thinking, and innovative capacities, thereby equipping them comprehensively for future challenges. The adoption of this paradigm is instrumental in nurturing a generation of learners characterized by adaptability, creativity, and competitiveness, essential for navigating the complexities of future societal demands.

Looking ahead, the field of social learning research holds vast potential, yet several critical areas demand immediate exploration. Primarily, the developmental model of selective learning across diverse cultural contexts warrants further investigation. Such research would elucidate the profound influence of cultural factors on individual learning strategies, offering a scientific foundation for cross-cultural educational practices and fostering international educational collaboration. To advance our understanding of selective learning’s developmental trajectory across cultures, we propose implementing cross-cultural longitudinal studies. Employing mixed-methods approaches will elucidate how cultural factors shape individual learning strategy development. Secondly, a thorough analysis of the internal mechanisms of selective learning among children of varying ages is crucial. This analysis is expected to uncover the effects of additional variables such as emotional states and social relationships on learning preferences, thereby informing the development of more precise and effective educational intervention strategies tailored to children’s individualized learning requirements. To comprehensively investigate the internal mechanisms of selective learning across developmental stages, we propose integrating experimental paradigms with neuroimaging techniques. Analyzing how variables like emotional states and social relationships shape learning preferences will enable the development of precisely targeted educational interventions tailored to individual learning profiles.

Additionally, the accelerating pace of educational technology innovation highlights its growing role in facilitating social learning. Emerging technologies, including online education platforms and virtual reality environments, offer novel avenues for observing and emulating behaviors, potentially revolutionizing individual learning choices and strategies. Future research should concentrate on how these technologies influence selective learning, aiming to refine the design and application of educational technologies to better support personalized learning and enhance educational quality and efficacy. To investigate how emerging educational technologies shape selective learning, we recommend empirical studies utilizing online education platforms and virtual reality environments. Systematic observation and analysis of children’s interaction patterns with these technologies will enable the refinement of their design and implementation. This approach will optimize educational technology to better support personalized learning pathways and enhance instructional efficacy.

The convergence of educational practice with social learning theory represents a pivotal trend for the future, necessitating a dynamic feedback loop between the two. Educators must transcend their traditional role as knowledge disseminators to become astute observers and researchers of the learning process. Enhancing educational effectiveness hinges on meticulous attention to students’ learning behaviors in the classroom, reflective adaptation based on selective learning theories, and the continuous refinement of teaching methodologies to accommodate the evolving and diverse needs of students.

In our view, future research might adopt a multi-dimensional perspective, comprehensively considering cross-cultural differences, individual variability, and the innovative application of educational technologies. Such an approach will holistically enhance children’s selective learning abilities in educational settings. This endeavor not only aids in improving children’s immediate learning outcomes but also establishes a robust foundation for their future learning and life skills, enabling them to navigate and thrive in an ever-evolving society with greater adaptability and competitiveness.

## Data Availability

The original contributions presented in the study are included in the article/supplementary material, further inquiries can be directed to the corresponding authors.
